# Time-Embedding Transformer-Based Remaining Lifetime Prediction of Organic Coatings: From Short-Term EIS Monitoring to Long-Term Performance Evolution

**DOI:** 10.3390/ma19214711

**Published:** 2026-11-05

**Authors:** Ziqiang Li, Suhang Hu, Fangnian Wang, Wei Hu, Yuming Tang, Xuhui Zhao, Yu Zuo

**Affiliations:** 1 Key Laboratory of Carbon Fiber and Functional Polymer, Ministry of Education, Beijing University of Chemical Technology, Beijing 100029, China; l18797812485@163.com (Z.L.); zuoy@mail.buct.edu.cn (Y.Z.); 2 School of Information Science and Technology, Beijing University of Chemical Technology, Beijing 100029, China; husuhang26@stu.pku.edu.cn (S.H.); wfnian@gmail.com (F.W.); huwei@mail.buct.edu.cn (W.H.); 3 Institute of Medical Technology, Peking University Health Science Center, Peking University, Beijing 100191, China

**Keywords:** organic coating, EIS, lifetime prediction, transformer deep learning, time-series data

## Abstract

The long-term degradation of organic coatings is governed by coupled effects of material and environmental factors, which poses great difficulties for accurate service performance prediction. Electrochemical impedance spectroscopy (EIS) is a common technique for evaluating coating aging and failure. However, conventional EIS empirical models have poor generalization and limited capacity to describe dynamic degradation processes, while existing data-driven neural networks cannot effectively capture temporal dependencies or support reliable long-term extrapolation. To solve these problems, this study develops a modified time-embedding transformer (TET) model to predict coating degradation and remaining service life using only EIS data available up to the prediction time. The embedded time-embedding module enhances temporal feature extraction, and the self-attention structure captures long-range dependencies in impedance time series. The low-frequency impedance modulus (|*Z*|_0.01 Hz_) was adopted as the primary model input, and the model was built and checked separately for nine epoxy coating–environment combinations. The results demonstrate that, when each coating system was modeled and checked separately, the TET model presents high prediction accuracy and stability across the studied systems. Compared with typical neural networks, the proposed method reduces prediction errors and effectively overcomes the limitations in dynamic degradation description and long-term trend extrapolation. This study provides an efficient and accurate method for coating durability evaluation to support the maintenance decision-making of anticorrosive organic coatings.

## 1. Introduction

Organic coatings represent one of the most important means of metal surface protection, playing a crucial role in enhancing the corrosion resistance of metallic materials, extending their service life, and ensuring functional safety. However, during service, coatings undergo irreversible degradation under the influence of multiple factors, including environmental media, ultraviolet radiation, and microbial activity. This process is highly complex and closely related to various factors such as coating chemical composition, environmental conditions, and the state of the underlying metal [1]. Over time, coating performance gradually deteriorates and protective efficacy diminishes, thereby threatening the durability and safety of the equipment. Therefore, accurate assessment of the evolution of coating protective performance and prediction of its remaining lifetime are of great significance for optimizing equipment maintenance strategies, reducing life-cycle costs, and ensuring the safe operation of facilities. In particular, for long-life protective coatings, accurately predicting their long-term behavior at an early stage presents a major challenge.

The performance evaluation of coatings typically relies on aging tests, in which the degree of coating degradation is analyzed by monitoring changes in physicochemical or electrochemical parameters. Common aging tests include natural exposure and indoor accelerated aging, such as salt spray, UV irradiation, and damp-heat tests. Macroscopically, coating degradation manifests as gloss loss, color change, chalking, blistering, cracking, and reduced adhesion; microscopically, electrochemical performance is reflected in decreased impedance or open-circuit potential decay. Among these, electrochemical impedance spectroscopy (EIS) is widely used in coating research owing to its simplicity, non-destructive nature, and quantitative characteristics. Parameters such as coating resistance and capacitance obtained from EIS data analysis can directly reflect the barrier performance of the coating and the corrosion state of the substrate [2,3]. Based on this, many researchers have constructed theoretical mathematical models incorporating parameters such as low-frequency impedance modulus, dielectric constant, coating thickness reduction, and adhesion strength to predict lifetime [4,5,6,7,8,9,10,11]. However, due to the complexity, diversity, and dynamic variations in coating materials and application environments, mechanism-based theoretical models often suffer from insufficient parameter generalization, limited experimental validation data, and difficulty in simulating dynamic processes, thus restricting the accuracy and applicability of their prediction results. Consequently, data-driven modeling approaches have garnered increasing attention from researchers.

Furthermore, with the continuous emergence of new coating products, traditional aging test methods, characterized by excessively long testing periods (typically more than six months), can no longer meet the urgent demand for the rapid evaluation of coating performance and swift prediction of service life. The ability to effectively deduce long-term performance evolution trajectories from limited short-term test data, thereby enabling lifetime prediction, holds significant practical value.

In recent years, rapid advancements in artificial intelligence have made artificial neural networks (ANNs) efficient data-driven modeling tools, which have been widely applied in the field of material evaluation and prediction. Early efforts adopted classical machine-learning and shallow neural networks for EIS-oriented coating-degradation research. Self-organizing map (SOM) networks enable automatic degradation-stage classification from EIS signatures, though they are mainly suited for identifying present-day coating conditions and require abundant complete experimental datasets [12,13]. Tian et al. constructed backpropagation artificial neural networks (BP-ANNs) to establish nonlinear correlations among environmental factors, EIS features and service lifetime, yet these static fully connected architectures are not explicitly designed to capture temporal dependencies within sequential aging measurements [14]. Statistical degradation models and feed-forward neural networks have also been adopted for coating lifetime assessment. For such approaches, reliable prediction outcomes typically rely on complete time-series measurements covering the whole degradation-to-failure process [15,16,17]. Recent advances in deep learning have facilitated automated EIS interpretation: Bongiorno et al. reported dense-neural-network-based workflows for equivalent-circuit topology classification and parameter fitting, and artificial intelligence driven analytical platforms support high-throughput processing predominantly for individual static impedance spectra [18,19]. Transformer-family architectures have also found coating-related applications, for instance, Swin-transformer for scanning electron microscopy micro-crack super-resolution recognition on coating images, focusing on visual feature extraction rather than electrochemical time-series forecasting [20]. Furthermore, mechanism-guided piecewise multi-stage degradation models offer interpretable physical descriptions for coating failure processes, showing optimal performance when measurements covering major degradation phases are accessible [21]. Collectively, existing approaches have yielded valuable insights for coating performance evaluation, whereas dedicated deep-learning solutions tailored to extrapolate long-term protective evolution from limited short-term EIS sequential records remain comparatively limited.

To address these challenges, this work proposes a modified time-embedding transformer (TET). Taking the low-frequency impedance modulus as the key input, this model aims to extract implicit patterns from limited short-term data and infer the evolution of coating performance over future time periods. Through comparative analysis with various modern neural network models, the significant advantages and robustness of the TET model in coating lifetime prediction are demonstrated.

## 2. Corrosion Experimental Methods

### 2.1. Coating Specimen Preparation and Corrosion Experiments

A series of accelerated corrosion laboratory tests were carried out to characterize the degradation behavior of organic coatings under different corrosive conditions. Nine representative coating–environment combinations, covering three epoxy coating systems and three corrosive environments, were selected and systematically tested. The full time-series EIS data obtained from these nine groups of experiments constitute the fundamental and reliable dataset for model building and independent validation of the coating lifetime prediction model proposed in this work.

The substrate used was low-alloy steel (Bohai Steel Group, Tianjin, China), with specimen dimensions of 150 mm × 75 mm × 2.5 mm. The substrate surfaces were first pretreated to remove contaminants and scale: sandblasting to Sa2.5 grade (blast-cleaning, nearly free of scale and rust with only sparse spot-like residues, ISO 8501-1 [22] or manual mechanical grinding to St3 grade (hand- and power-tool (Bosch Power Tools (China) Co., Ltd., Hangzhou, China) cleaning removing loose rust to reveal distinct metallic lustre, ISO 8501-1) was performed. The surfaces were then wiped with ethanol (Shanghai Macklin Biochemical Co., Ltd., Shanghai, China) to thoroughly remove residual grease. Finally, the specimens were left to dry naturally in air prior to use.

Three typical epoxy coating systems were as follows: (1) Waterborne epoxy primer (Penguard Express WF, Jotun Coatings (Zhangjiagang, China) Co., Ltd.): a two-component system, with Component A mainly consisting of novel epoxy resin and propyltrimethylsilane, and Component B being a 3-aminopropyldiethylamine curing agent; the mass ratio of A to B was 2.5:1. (2) Solvent-Free universal epoxy primer (Jotun Coatings (Zhangjiagang, China) Co., Ltd.): a two-component polyamine-cured epoxy system, with a volume ratio of A to B of 2.5:1; Jotun Thinner (Jotun A/S, Sandefjord, Norway) was added during application. (3) Nano-modified epoxy primer (Zhongke Nano Coatings Technology (Suzhou, China) Co., Ltd.): Component A consisted of modified epoxy resin, nano-modifiers (including silica and nano-alumina), and anti-rust pigments, Component B was a modified amine curing agent; the mass ratio of A to B was 4:1. The coatings were applied onto the prepared steel specimens using high-pressure airless spraying. All specimens were cured at room temperature for 15 days to ensure complete crosslinking. Dry film thickness was measured using a TT230 coating thickness gauge (Time Group Inc., Beijing, China) and controlled within the range of within 80 ± 10 μm.

The prepared coated specimens were placed under different laboratory-accelerated corrosion environments for testing, resulting in nine coating–environment combinations (as shown in Table 1). Neutral salt-spray (NSS) tests were performed following ISO 9227:2022 [23]. For each experimental condition, at least six parallel specimens were set to ensure statistical reliability of the data.

### 2.2. Performance Testing Methods

Periodic impedance monitoring of the coated specimens was performed using a PARSTAT 2273 electrochemical workstation (Princeton Applied Research, Oak Ridge, TN, USA). A classical three-electrode system was employed: the working electrode was the coated specimen to be tested (with an exposure area of 20 cm^2^), the reference electrode was a saturated calomel electrode (SCE), and the counter electrode was a platinum electrode. Measurements were conducted under open-circuit potential (OCP) using 3.5% NaCl (Shanghai Aladdin Biochemical Technology Co., Ltd., Shanghai, China) solution (room temperature) as the electrolyte. The excitation signal was a sinusoidal wave with an amplitude of 10 mV, and the scanning frequency range was from 10^−2^−10^5^ Hz.

According to the national standard GB/T 5210-2006 [24], pull-off adhesion between the coating and the substrate was measured using a PosiTest Pull-Off Adhesion Tester (DeFelsko, Inc., Ogdensburg, NY, USA). Prior to testing, a standard test dolly with a diameter of 20 mm was bonded to the coating surface using a Ergo1690 (Kisling AG, Wetzikon, Switzerland) two-component methyl methacrylate structural adhesive and cured at room temperature for 24 h. During testing, a vertical pull was applied at a loading rate of 0.2 MPa/s. Six measurement points were randomly selected on each specimen, and the final result was calculated as the arithmetic mean.

During the experimental period, macroscopic morphology inspections were conducted regularly, and a digital camera (Canon Inc., Tokyo, Japan) was used to document failure characteristics such as blistering, rusting, and cracking on the coating surface. Additionally, a high-precision digital multimeter (Fluke Corporation, Everett, WA, USA) was employed to monitor the open-circuit potential of both bare steel and coated specimens over time, enabling a comprehensive assessment of the protective capability of the coating on the underlying metal.

## 3. Construction of Coating Lifetime Prediction Model Based on TET

### 3.1. Data Augmentation and Temporal Reconstruction Strategy

The raw dataset for the lifetime prediction model adopts time-series low-frequency impedance modulus |*Z*|_0.01 Hz_ (Ω⋅cm^2^) as its input variable, and test time (day) serves as the temporal index. Data are obtained from nine coating–environment combinations listed in Table 1. Each test condition includes six to ten parallel specimens, which were monitored starting from 2 h after exposure until coating failure, and the maximum monitoring duration reaches 414 days. Invalid outliers were eliminated from the raw measurement sequences before further processing.

Each coating–environment system was modeled independently. For a given system, the parallel specimens were split into a model-building group and an independent checking group. The model-building group was used for model development: its |*Z*|_0.01 Hz_ records were sorted chronologically by exposure time, with the first 80% employed for model fitting and the subsequent 20% reserved to verify model stability during training. The independent checking group comprised the remaining parallel specimens from the same coating–environment system (e.g., the same salt-spray condition). These specimens received no data augmentation and were excluded from all stages of model construction; this dataset remained completely unseen throughout model development and was used solely to evaluate the model’s predictive performance. No specimen was allocated to both groups, and data from different coating systems were strictly separated.

**Figure 1:**
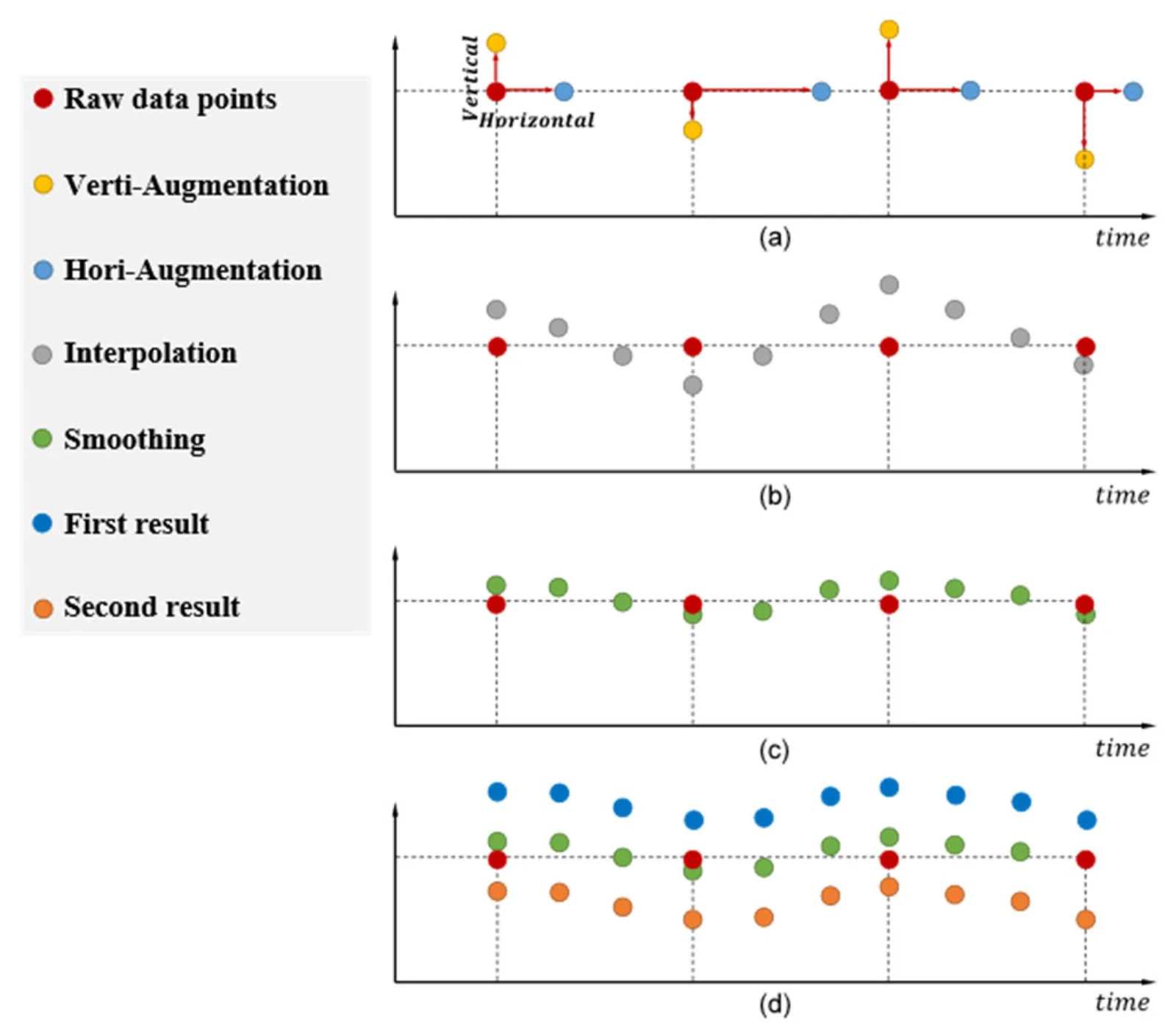
Schematic diagram of data augmentation: (**a**) horizontal and vertical augmentation; (**b**) results after interpolation; (**c**) results after data smoothing; (**d**) results of two additional sets of data augmentation.

Obtaining long-term EIS monitoring data is costly and time-consuming, so the original experimental dataset suffers from limited sample quantity, which poses a major challenge for the time-series prediction framework. In this framework, the model takes 30 consecutive |*Z*| monitoring points to predict the subsequent time point and performs stepwise extrapolation. Training deep neural networks directly on such data may lead to overfitting or underfitting and degrade the model’s generalization. To address this problem, a four-step data augmentation and temporal reconstruction strategy was proposed in this study (Figure 1). This strategy improves dataset quality by enriching sample diversity, filling temporal gaps and suppressing random noise, allowing the model to effectively learn the representative features of coating degradation.

(1) Horizontal Augmentation

To enrich the feature distribution of samples, the convex-set-combination-based approach was adopted to generate synthetic samples. Assuming the original impedance data sequence is denoted as {*x*_1_, *x*_2_, ⋯, *x_n_*}, a new sample *x*^′^ was generated via random linear interpolation:

(1) $$\begin{array}{cc} x^{\prime }=\lambda x_{i}+\left(1-\lambda \right)x_{j} & 0\leq \lambda \leq 1 \end{array}$$

where *x_i_* and *x_j_* are two randomly selected distinct sample points from the sequence, and *λ* represents a random number following a uniform distribution. This operation constructs a continuous manifold between the original samples within the feature space and broadens the coverage of the model-building data.

(2) Vertical Augmentation

To simulate environmental fluctuations encountered in practical measurements and improve model robustness, Gaussian white noise was superimposed onto original measured data:

(2) $$x^{\prime \prime }=\begin{array}{cc} x+\alpha \cdot & N\left(0,\sigma^{2}\right) \end{array}$$

where *N*(0,*σ*^2^) denotes a normal distribution with a mean value of zero and a standard deviation of *σ*, and *α* is the noise-scaling factor that adjusts the perturbation amplitude to guarantee the physical rationality of synthetic outputs.

(3) Interpolation

To handle inconsistent sampling intervals and missing-value issues, linear interpolation together with quadratic spline interpolation was utilized to densify the time points. The formula for linear interpolation is expressed as

(3) $$y=y_{1}+\frac{\left(x-x_{1}\right)\cdot \left(y_{2}-y_{1}\right)}{x_{2}-x_{1}}$$

The formula for quadratic spline interpolation is given below:

(4) $$y=y_{1}\frac{\left(x-x_{2}\right)\left(x-x_{3}\right)}{\left(x_{1}-x_{2}\right)\left(x_{1}-x_{3}\right)}+y_{2}\frac{\left(x-x_{1}\right)\left(x-x_{3}\right)}{\left(x_{2}-x_{1}\right)\left(x_{2}-x_{3}\right)}+y_{3}\frac{\left(x-x_{1}\right)\left(x-x_{2}\right)}{\left(x_{2}-x_{1}\right)\left(x_{3}-x_{2}\right)}$$

where *x* is the target time point for interpolation and *y* stands for the calculated impedance value. By interpolation, the non-uniform discrete sequences were converted into equally spaced time-series data satisfying the input format requirements of the model.

(4) Smoothing

To mitigate interference from high-frequency random noise, a moving-average algorithm was applied for sequences smoothing. For an original sequence with the length of *n*, *x* = {*x*_1_, *x*_2_, ⋯, *x_n_*}, the smoothed value of the *i*-th point under a window length *m* is defined as

(5) $$x^{\prime }=\begin{array}{cc} \frac{1}{m}\sum_{i-m+1}^{i}x_{j} & m\leq i\leq n \end{array}$$

After the above four-step processing, the dataset features expanded sample diversity, complete uniform time sequences and reduced random noise, providing a well-processed dataset for subsequent model training.

### 3.2. Time-Embedding Transformer (TET) Model Architecture

**Figure 2:**
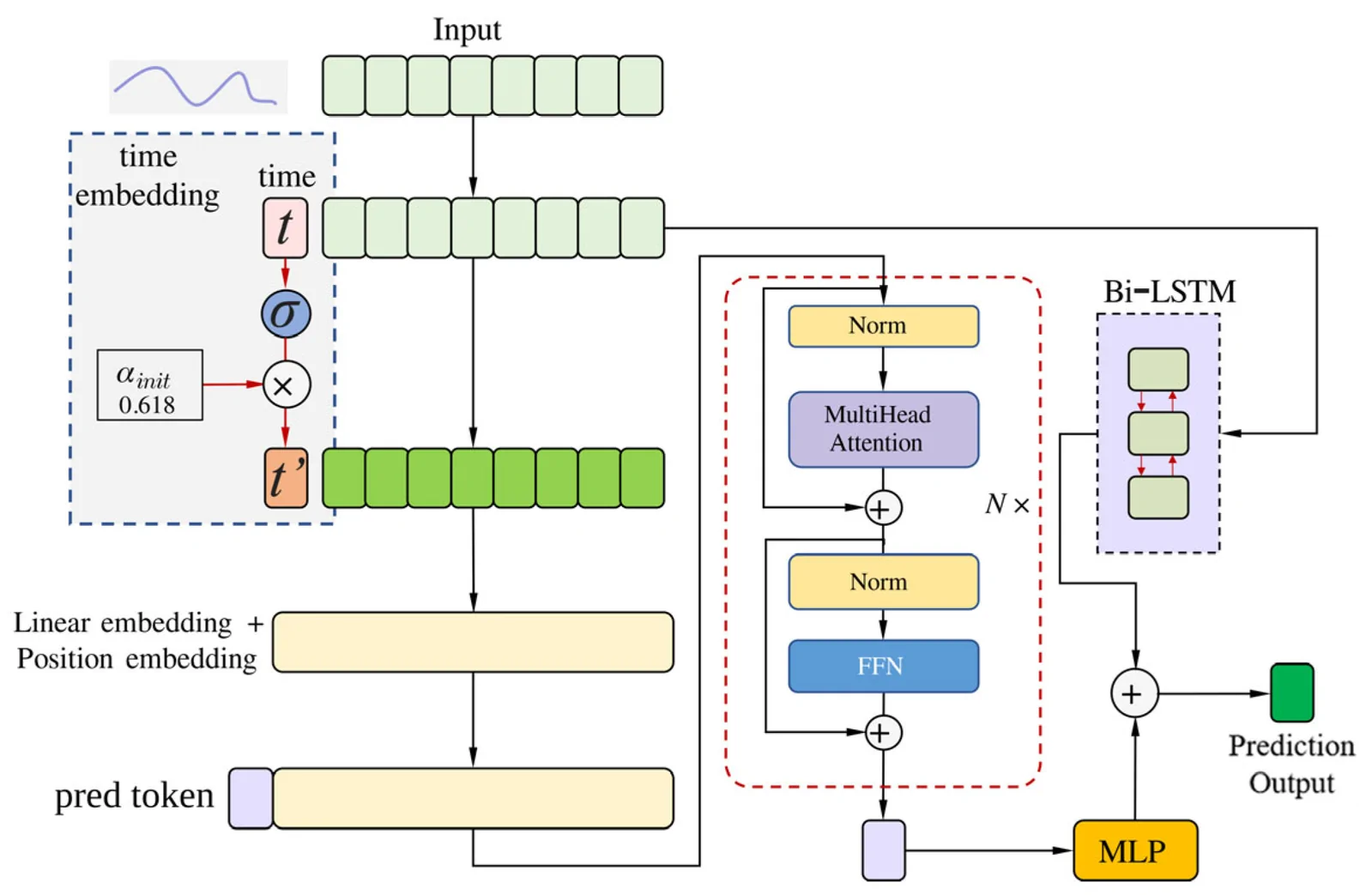
Schematic diagram of the time-embedding transformer architecture for coating lifetime prediction.

The TET model proposed in this study is a hybrid deep learning architecture specifically designed for coating lifetime prediction. As illustrated in Figure 2, the model adopts a dual-branch parallel structure: the main branch consists of a time-embedding module and a transformer encoder, aiming to capture global long-range dependencies within the sequence; the auxiliary branch employs a Bidirectional Long Short-Term Memory (Bi-LSTM) network, focusing on extracting local temporal dynamic features. The outputs of the two branches were concatenated and then fused through a fully connected layer (FC) to generate the final prediction:

*Y*_pred_ = *FC*([*H*_Trans_; *H*_Bi-LSTM_]) (6)

where *FC* denotes the fully connected layer, [;] represents the vector concatenation operation, and *H*_Trans_ and *H*_Bi-LSTM_ are the feature outputs of the main and auxiliary branches, respectively.

#### 3.2.1. Time-Embedding Module

Conventional positional encoding only reflects sequence order and is inadequate for representing the non-uniform time spans characteristic of coating-degradation processes. The time-embedding module introduces a learnable time-sensitivity mechanism that encodes absolute temporal information into the input features. The specific implementation is as follows: a learnable scalar parameter *α* (initialized with the golden ratio of 0.618 to provide a stable convergence starting point) was introduced, and the time stamp *t* was converted into a time-embedding vector *E*_t_ through a nonlinear mapping function:

*E*_t_ = *tanh*(*α* ⋅ *t*) (7)

During model building, *α* was dynamically updated through gradient backpropagation, enabling the model to adaptively learn the weighted influence of time intervals on coating performance evolution, thereby capturing dynamic changes in degradation rates with greater precision.

#### 3.2.2. Transformer-Encoding Module

As the core global feature extractor of the model, the transformer module employs a multi-head self-attention (MHSA) mechanism to establish correlations between any two points in the sequence, effectively addressing the vanishing gradient problem in long sequences.

Self-attention mechanism: Attention weights for the query, key, and value matrices were computed to capture long-range dependencies during impedance evolution.

Encoder structure: Considering the small-sample characteristics, only the encoder portion of the transformer was adopted in this study, consisting of multiple stacked layers of MHSA and feed-forward neural networks (FFNs), with residual connections and layer normalization introduced to prevent overfitting.

Learnable positional encoding: In addition to time-embedding, learnable positional encoding vectors were superimposed to preserve relative order information within the sequence.

#### 3.2.3. Bidirectional LSTM (Bi-LSTM) Auxiliary Module

To compensate for potential deficiencies in the transformer in extracting local fine-grained features, a Bi-LSTM was introduced as an auxiliary branch. The specific design is as follows:

Bidirectional structure: Comprising two independent LSTM layers—forward and backward—that scan the sequence from past to future and future to past, respectively, with their outputs concatenated as

*H*_Bi-LSTM_ = [LSTM_forward_(*X*); LSTM_backward_ (*X*)] (8)

where *X* is the input sequence.

Gated memory mechanism: The core of the LSTM is a cell state vector that stores long-term memory, where information flow is regulated by three gating mechanisms: input gate, forget gate, and output gate. This structure allows the bidirectional LSTM to preserve early information and reuse it in subsequent calculations, enabling the capture of dynamic evolution of coating performance.

The TET backbone adopted in this work features a 256-dimensional embedding, six transformer layers, four attention heads each with a head dimension of six, a 256-dimensional feed-forward network, zero dropout, GELU activation, pre-layer normalization, and CLS-token pooling. The model was trained using the Adam optimizer with an initial learning rate of 0.001, MSE loss, a batch size of 1024, a maximum of 10 epochs, early stopping with a patience of three, and a random seed of 42. The learning rate was reduced at epochs two, four, six, and eight.

### 3.3. Coating Remaining Lifetime Prediction Workflow Based on TET

Based on the trained TET model, the coating lifetime prediction workflow designed in this study consists of the following four steps: baseline curve mapping, interpolation and model inference, iterative prediction, and lifetime assessment.

(1) Baseline Curve Mapping and Input Construction

Data acquisition: The low-frequency impedance modulus |*Z*|_0.01 Hz_ of the target coating at the current time *t*_current_ was acquired.

Baseline fitting: Multiple sets of parallel experimental data for the same coating system under the same environment were retrieved from the historical database, and a reference baseline degradation curve was constructed using polynomial fitting.

At each prediction origin, only data acquired up to that time were used to construct the baseline; later observations were not included.

Input sequence generation: By combining the real-time monitoring point with the baseline curve trend, a standardized input sequence of length *L* = 30, *Z*_input_ = {*Z*_1_,*Z*_2_,⋯,*Z*_30_}, was generated as the initial state for model inference.

(2) Temporal Interpolation and Single-Step Inference:

Dimensional alignment: The model takes 30 consecutive monitored |*Z*| values together with the exposure time as input. When fewer than 30 measurements were available, quadratic interpolation was applied to reach the required length of 30 points.

Model inference: The interpolated sequence was input into the TET model to predict the impedance *Z_t_*_+1_ at the next time point.

(3) Rolling Iterative Prediction

Sequence update: The predicted value *Z*_t+1_ was incorporated into the time series, and the earliest data point was removed, forming a new sliding window sequence [*Z*_2_, *Z*_3_, …, *Z_t_*_+1_].

Recursive extrapolation: The “interpolation–inference–update” process was repeated to progressively generate the future impedance evolution trajectory until the sequence length covered the preset evaluation period or reached the failure threshold.

(4) Remaining Lifetime Assessment

Failure criterion: A commonly adopted reference value for coating failure impedance is 1 × 10^6^ Ω cm^2^, and alternative thresholds may also be defined following relevant engineering standards. In this work, group-specific *Z*_crit_ values were determined by integrating multiple experimental failure indicators.

Lifetime calculation: The time *t*_fail_ at which the predicted curve first dropped to *Z*_crit_ was located. The remaining lifetime of the coating *t*_remaining_ was then calculated as

*t*_remaining_ = *t*_fail_ − *t*_current_ (9)

## 4. Results and Analysis

### 4.1. Analysis of Corrosion Experimental Results

In this study, the degradation process and evolution patterns of organic coatings under corrosive environments were systematically elucidated through electrochemical impedance spectroscopy (EIS), pull-off adhesion testing, morphological observation, and open-circuit potential (OCP) monitoring. All experiments were terminated when significant blistering or rusting appeared on the specimen surfaces. Taking System 7 (nano-modified epoxy coating/3.5% NaCl solution immersion) in Table 1 as an example, the evolution of key performance parameters throughout the entire process from intact to failed coating was analyzed in depth, with emphasis placed on elucidating the quantitative relationship between the low-frequency impedance modulus (|*Z*|_0.01 Hz_) and the coating protection level, aiming to provide a physical basis for model construction that extrapolates long-term lifetime from short-term impedance data. Detailed data results and pattern analysis for System 1 can be found in previous work [6]. For the other coating–environment combinations, the variations in |*Z*|_0.01 Hz_ throughout the entire life-cycle are representatively presented.

**Figure 3:**
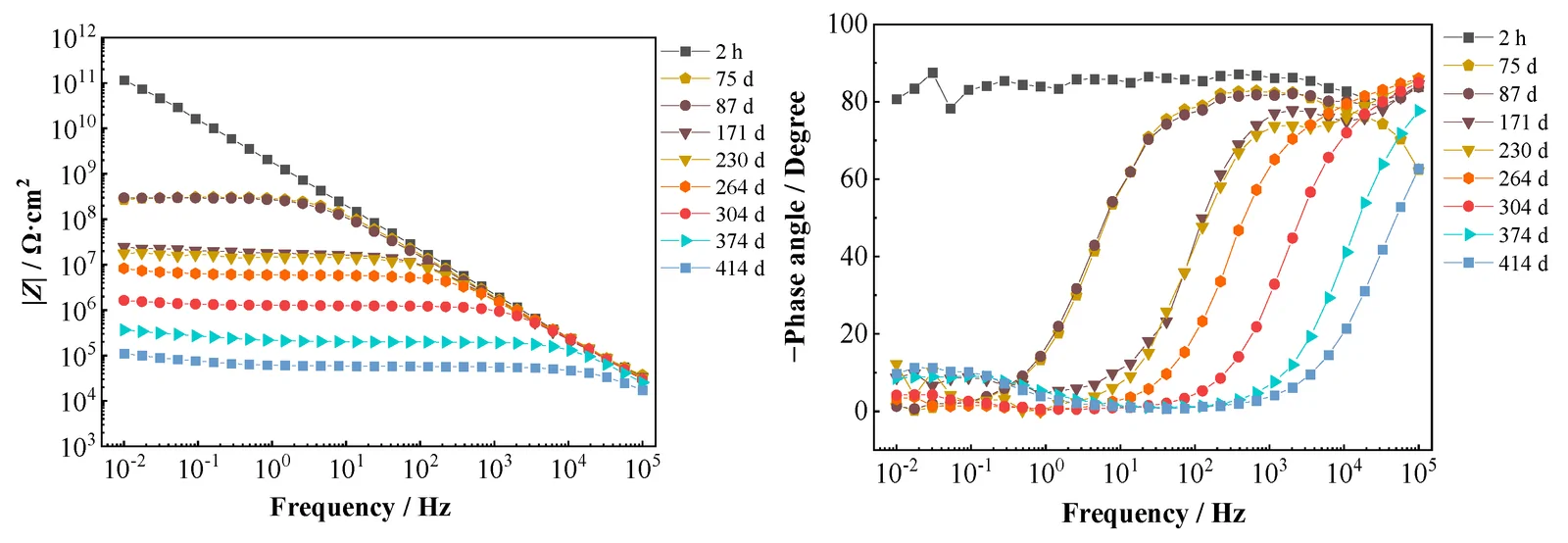
EIS spectral evolution of nano-epoxy coating in 3.5% NaCl solution.

**Figure 4:**
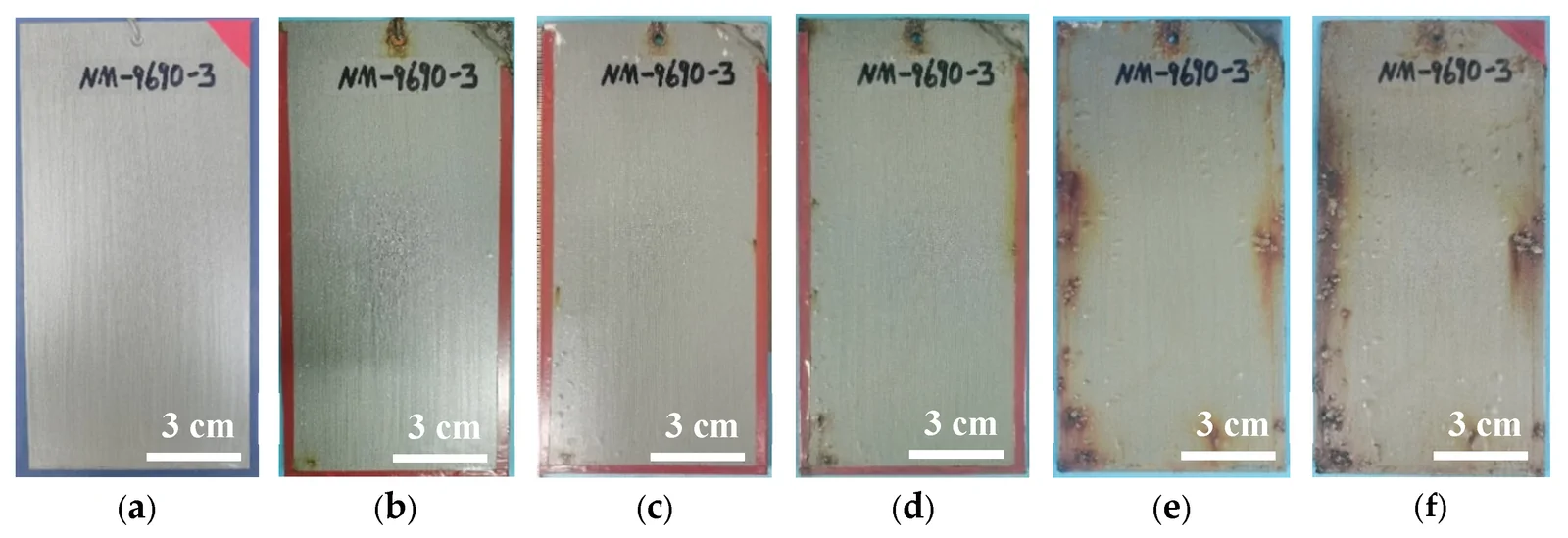
Surface macroscopic morphology photographs of nano-epoxy coating after different immersion periods in 3.5% NaCl solution: (**a**) 0 h, (**b**) 87 d, (**c**) 150 d, (**d**) 244 d, (**e**) 324 d, (**f**) 404 d.

Figure 3 shows the EIS spectral evolution of the nano-epoxy coating during immersion in 3.5% NaCl solution. At the beginning of the test (2 h), the specimen exhibited excellent barrier performance, with a |*Z*|_0.01 Hz_ modulus as high as 1 × 10^11^ Ω⋅cm^2^. The Bode phase angle ranged between 80° and 90°, indicating that the coating behaved as an ideal capacitor, being dense and defect-free. With prolonged immersion time, the electrolyte gradually penetrated the coating and reached the substrate interface, leading to a significant decrease in the impedance modulus and phase angle in the medium- and low-frequency regions, indicating decrease in the coating’s barrier performance. Combined with the macroscopic morphological observations in Figure 4, the coating surface underwent a process from intact to local blistering, followed by an increase in both the size and number of blisters accompanied by severe rusting: a small number of blisters appeared at the edges at approximately 150 d; by around 244 d, blisters expanded toward the central area with noticeable edge rusting; by 324 d, blisters in the central area had increased in size and number, with severe edge rusting, marking the near-complete loss of the coating’s protective function.

To investigate the correlation between electrochemical performance and mechanical properties, adhesion tests were performed simultaneously at time points corresponding to each order-of-magnitude decrease in |*Z*|_0.01 Hz_. Table 2 summarizes the adhesion test result at different experimental stages. The initial adhesion was 12.25 MPa, with a failure mode of 100% cohesive failure within the coating, corresponding to |*Z*|_0.01 Hz_ ≈ 10^11^ Ω cm^2^, indicating excellent interfacial bonding strength and intact coating density, effectively blocking corrosive media. As the electrolyte accumulated at the coating/substrate interface, hydrolysis reactions occurred, weakening interfacial bonding, and adhesion exhibited a decreasing trend, with the failure mode gradually transitioning from cohesive failure to interfacial delamination. Key data revealed that at 260 d, adhesion decreased to approximately 6.0 MPa (attenuation rate of ~51%), with the proportion of interfacial delamination area increasing to about 46%, while |*Z*|_0.01 Hz_ decreased to 1.0 × 10^7^ Ω cm^2^, and macroscopically, blisters at the specimen edges began to expand toward the central area. At 324 d, adhesion further decreased to 4.7 MPa (attenuation rate of ~62%), with the interfacial delamination area reaching 78%, while |*Z*|_0.01 Hz_ ≈ 2.0 × 10^6^ Ω⋅cm^2^, and the coating surface exhibited dense blistering and severe rusting. At 370 d, |*Z*|_0.01 Hz_ was 1.0 × 10^6^ Ω⋅cm^2^, adhesion decreased to 3 MPa (attenuation rate of ~76%), and the failure mode was predominantly interfacial delamination (>90%).

**Figure 5:**
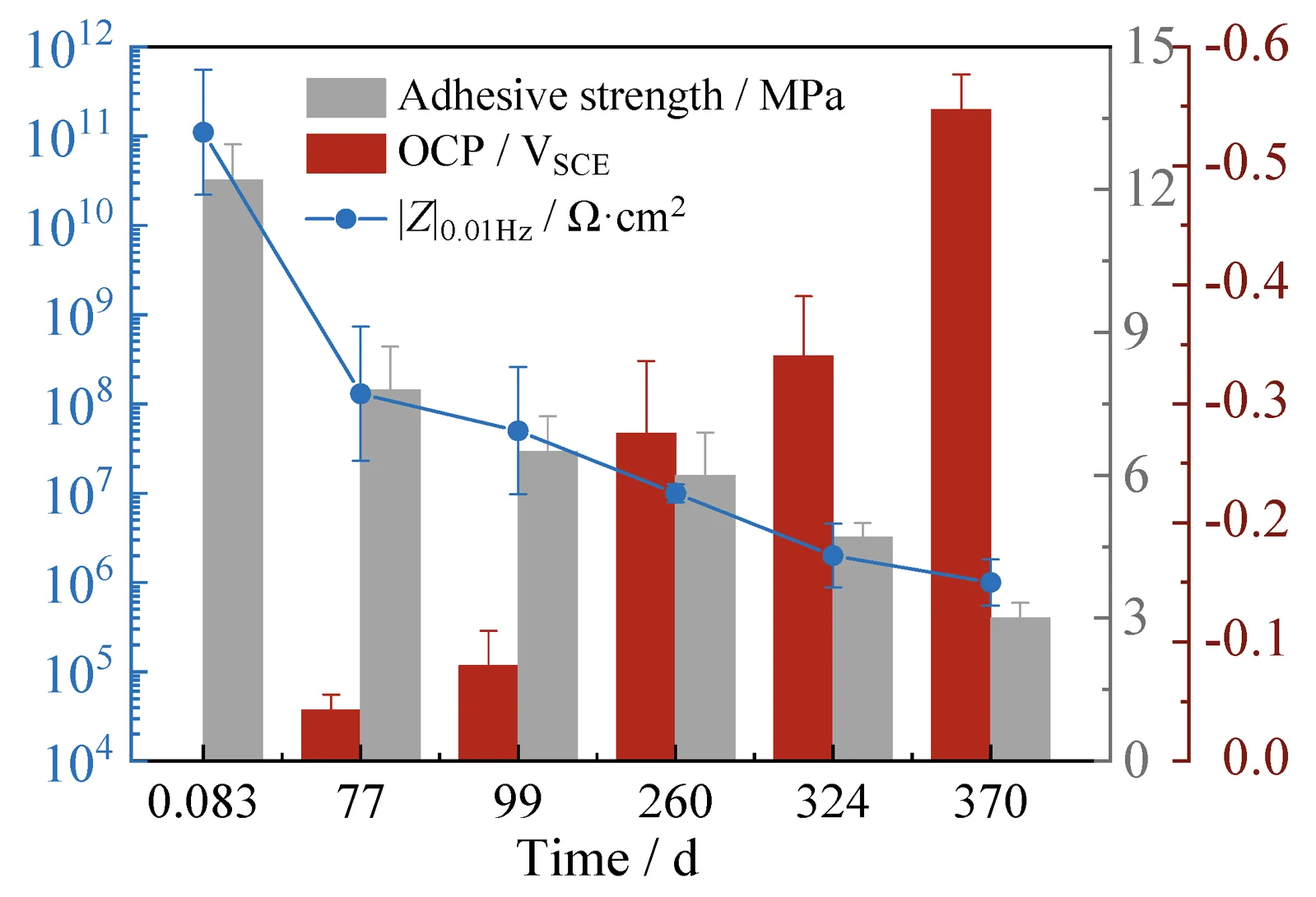
Adhesion, |*Z*|_0.01 Hz_, and OCP results for nano-epoxy coating in 3.5% NaCl solution.

Figure 5 visually demonstrates a positive correlation between |*Z*|_0.01 Hz_ and adhesion. Although no unified international standard currently defines an absolute threshold for coating adhesion failure, engineering experience often recommends maintenance when adhesion falls below 3–5 MPa accompanied by significant rusting. The results of this study indicate that when the adhesion attenuation rate reaches approximately 50%, the coating barrier performance is significantly degraded (|*Z*|_0.01 Hz_ ≈ 1 × 10^7^ Ω cm^2^, with visible blistering on the surface); when adhesion falls below 5 MPa with an attenuation rate exceeding 60%, the coating essentially loses its protective capability (|*Z*|_0.01 Hz_ ≈ 1 × 10^6^ Ω cm^2^, with severe surface blistering and rusting). This finding is highly consistent with previous research results [6] and further corroborates earlier literature: when the |*Z*|_0.01 Hz_ of a carbon steel/organic coating system falls below 1.0 × 10^6^ Ω⋅cm^2^, the coating no longer provides effective protection to the substrate [21,25].

To further validate the loss of coating protective function, the open-circuit potential of the specimens was monitored, as shown in Figure 5. With prolonged immersion time, OCP gradually shifted negatively. By 260 d, OCP decreased to −0.27 V_SCE_; by 324 d, it decreased to −0.34 V_SCE_; by 370 d, it decreased to approximately −0.55 V_SCE_, approaching the corrosion potential of the bare steel substrate (−0.59 V_SCE_), indicating that the coating had lost its barrier function. In summary, good consistency was observed among coating surface morphology, adhesion, OCP, and |*Z*|_0.01 Hz_, collectively forming a complete chain of evidence for evaluating coating service status and confirming the rationality and sufficiency of selecting |*Z*|_0.01 Hz_ as a single key input parameter to characterize the overall coating-degradation state.

**Figure 6:**
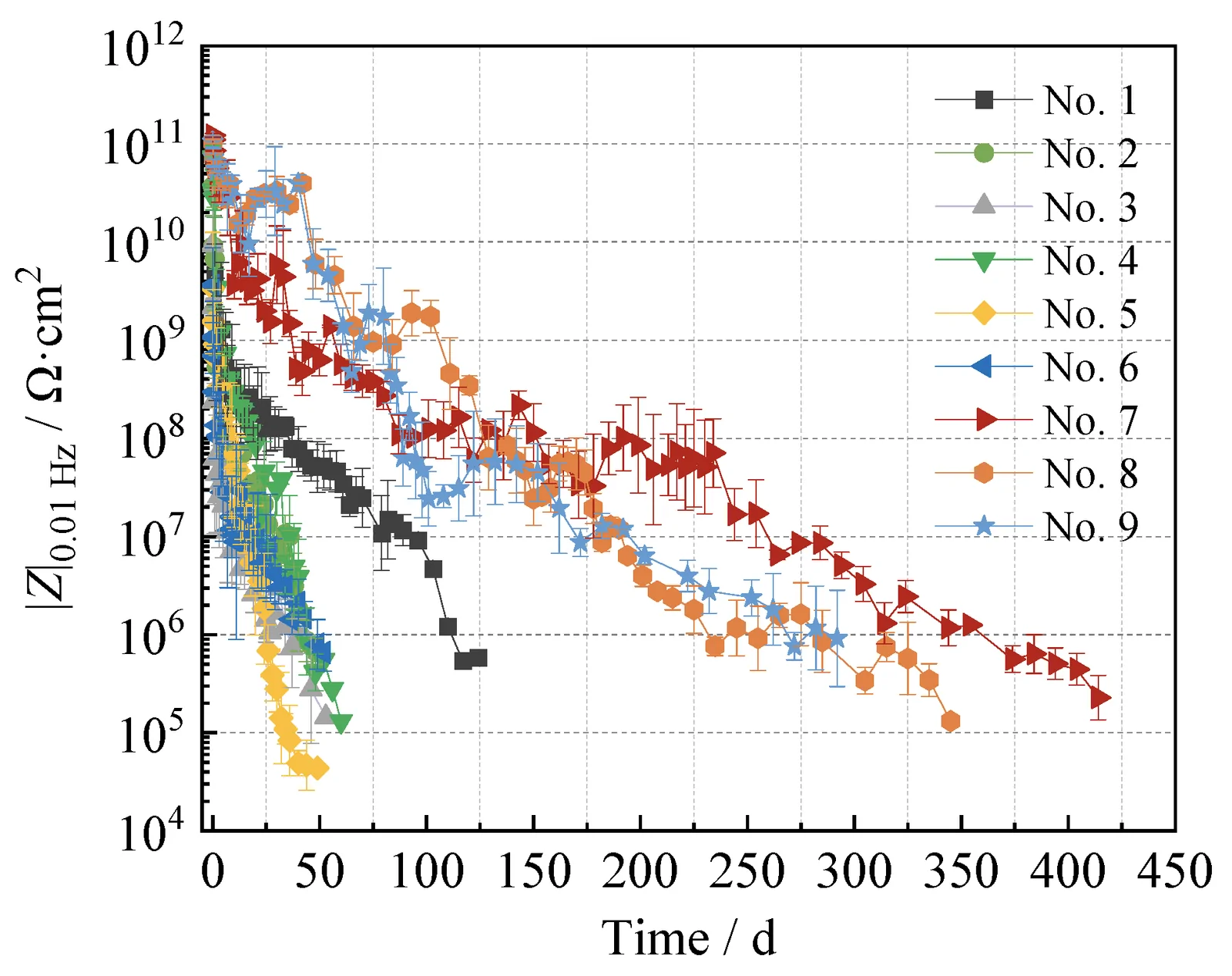
Evolution of |*Z*|_0.01 Hz_ with test time for nine different coating–environment combinations.

In electrochemical impedance spectroscopy analysis, the low-frequency impedance modulus (|*Z*|_0.01 Hz_) is widely recognized as a key indicator reflecting the barrier performance of organic coatings due to its high sensitivity to coating porosity and interfacial state [4,26,27]. During the natural degradation process of coatings, this parameter typically exhibits a monotonic decreasing trend and is amenable to continuous, quantitative monitoring. The corrosion experiments in this study further confirmed that across different protective coating systems (waterborne epoxy, solvent-free epoxy, and nano-modified epoxy) and under different environmental conditions (room temperature immersion, salt spray, and temperature cycling), the |*Z*|_0.01 Hz_ of all experimental groups presents an overall monotonic decreasing trend (as shown in Figure 6). This consistent macroscopic evolution pattern reflects the physical essence of medium permeation and interfacial debonding during coating failure and provides a reliable data foundation for utilizing data-driven models, such as TET, to learn degradation trajectories from short-term data and extrapolate long-term lifetime.

Notably, slight transient fluctuations can be observed in some test curves. Apart from experimental interferences such as unstable electrode contact and testing environment variations, local impedance variations may also originate from intrinsic coating–substrate physicochemical processes including water uptake, interfacial passivation and localized degradation. Small-amplitude fluctuations generally impose limited impacts on the construction of prediction models. Nevertheless, considerable or frequent data fluctuations may disturb the fitting of real coating-degradation trends and further impair model reliability. For this reason, the validity of experimental data is critical for obtaining reliable prediction models. Accordingly, given sufficient parallel specimens and high-frequency data collection, a small number of severely abnormal data points can be appropriately eliminated to rule out accidental experimental interference, so as to improve the stability and reliability of the established prediction model.

### 4.2. Data Augmentation Results

**Figure 7:**
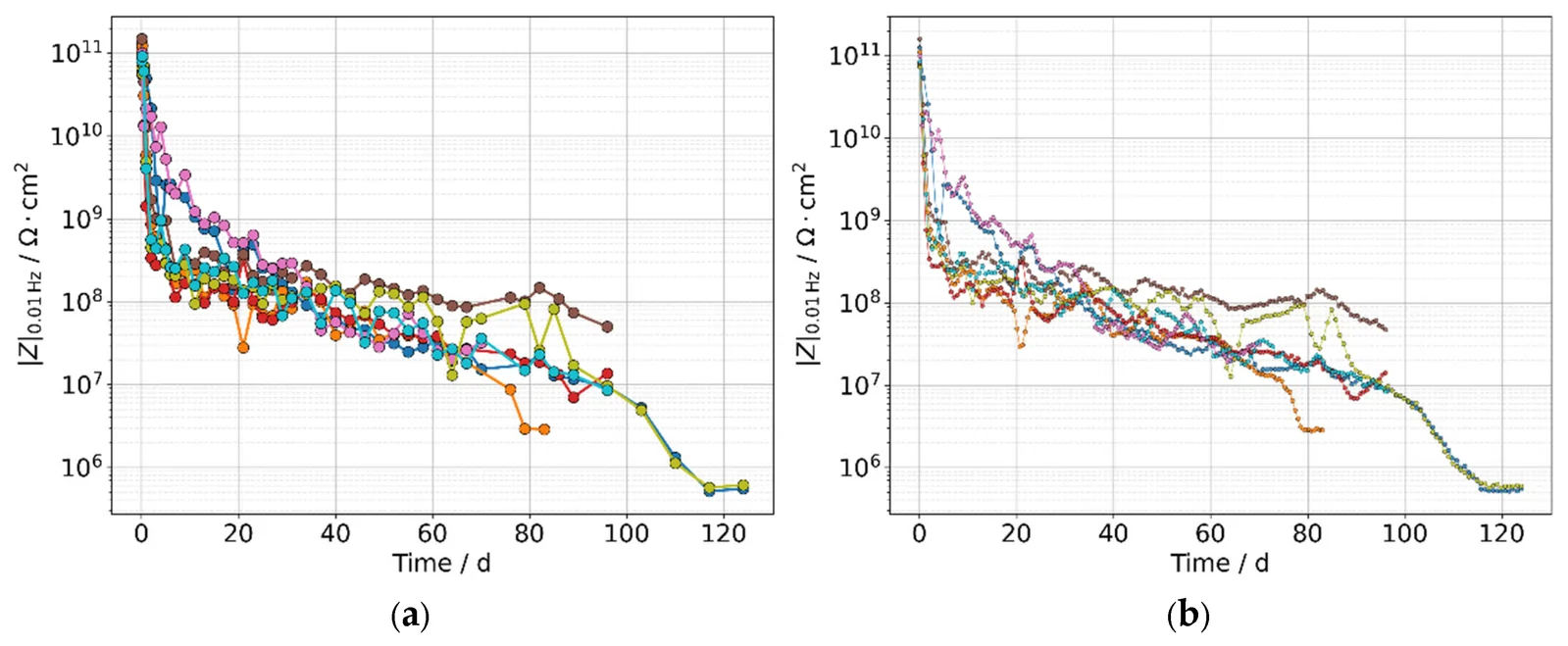
Comparison of real dataset augmentation effects: (**a**) original data, (**b**) high-density continuous sequence data after four-step augmentation.

As the key parameter characterizing coating degradation, the low-frequency impedance modulus (|*Z*|_0.01 Hz_) exhibits significant non-stationary characteristics in its time series: absence of obvious periodicity, non-uniform sampling intervals, and a strictly monotonic decreasing trend. To address this, the four-step data augmentation algorithm described in Section 3.1—comprising horizontal augmentation, vertical augmentation, multi-order interpolation reconstruction, and smoothing—was applied only to the model-building group before the 80/20 division within that group. The augmentation results are presented using the first coating system in Table 1 (solvent-free epoxy/3.5% NaCl) as an example. As shown in Figure 7, panel (a) displays the original data from six parallel specimens in the model-building group, revealing noticeable uneven sampling intervals and local fluctuations; panel (b) shows the augmented dataset. It can be observed that the augmented data not only filled temporal gaps to form high-density continuous sequences but also effectively suppressed random measurement noise while preserving the overall monotonic degradation trend of the coating. Furthermore, to address the issue of inconsistent sequence lengths arising from variations in failure times among different parallel specimens, a minimum common time window strategy (i.e., “shortest-sequence alignment”) was adopted. All model-building records were truncated to the shortest experimental duration, ensuring uniformity in the input matrix dimensions and providing a high-quality standardized dataset for model building.

### 4.3. Model Prediction Performance Evaluation and Analysis

#### 4.3.1. Evaluation Metrics and Checking Methods

To comprehensively quantify the accuracy of the time-embedding transformer (TET) model in coating performance extrapolation and lifetime prediction, a dual evaluation system encompassing curve morphological similarity and lifetime prediction accuracy was established:

(1) Curve Morphological Similarity Evaluation: Cosine similarity and Euclidean distance were employed to quantify the agreement between the predicted curves and the actual measurement data in terms of morphology and magnitude. Cosine similarity was used to characterize directional consistency in the variation trend (ranging from zero to one, with values closer to one indicating higher trend consistency), while Euclidean distance reflected the absolute deviation in magnitude between predicted and measured values (smaller values indicating higher fitting accuracy). During independent checking, low-frequency impedance modulus data from different service stages were selected as input windows to extrapolate future time series, and the complete generated sequences were used to calculate the above two metrics for comprehensive assessment of model prediction performance.
(2) Remaining Lifetime Prediction Accuracy Evaluation: To overcome the limitation of the single-impedance threshold method being susceptible to data fluctuations, a slope change point detection method was employed to assist in establishing the failure time *t*_fail_. Failure was defined as the point at which the impedance decline rate significantly accelerated (manifested as a sudden change in the second derivative) and the value fell below the critical range. On this basis, the relative error (RE) between the predicted failure time (*t*_pred_) and the actual failure time (*t*_real_) was calculated as RE = ∣*t*_pred_ − *t*_real_∣/*t*_real_ to quantitatively characterize the model accuracy in lifetime prediction tasks.

#### 4.3.2. Analysis of Typical System Prediction Results

**Figure 8:**
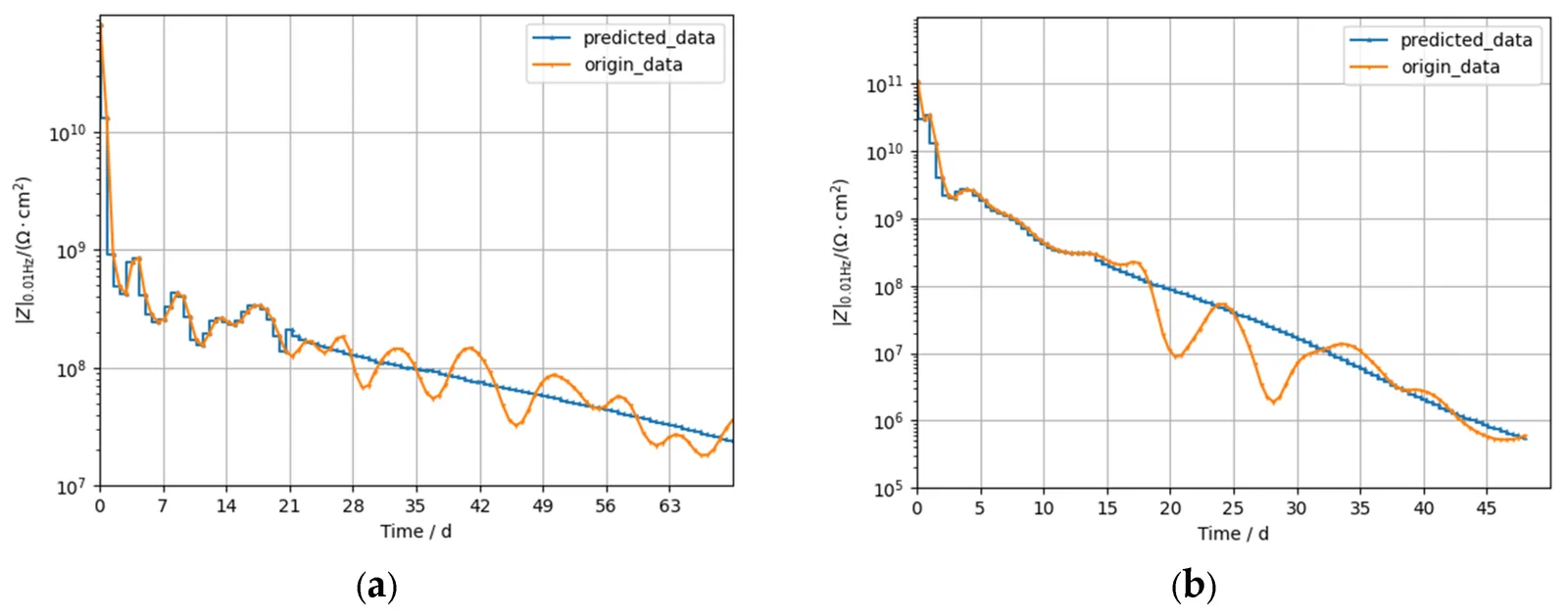

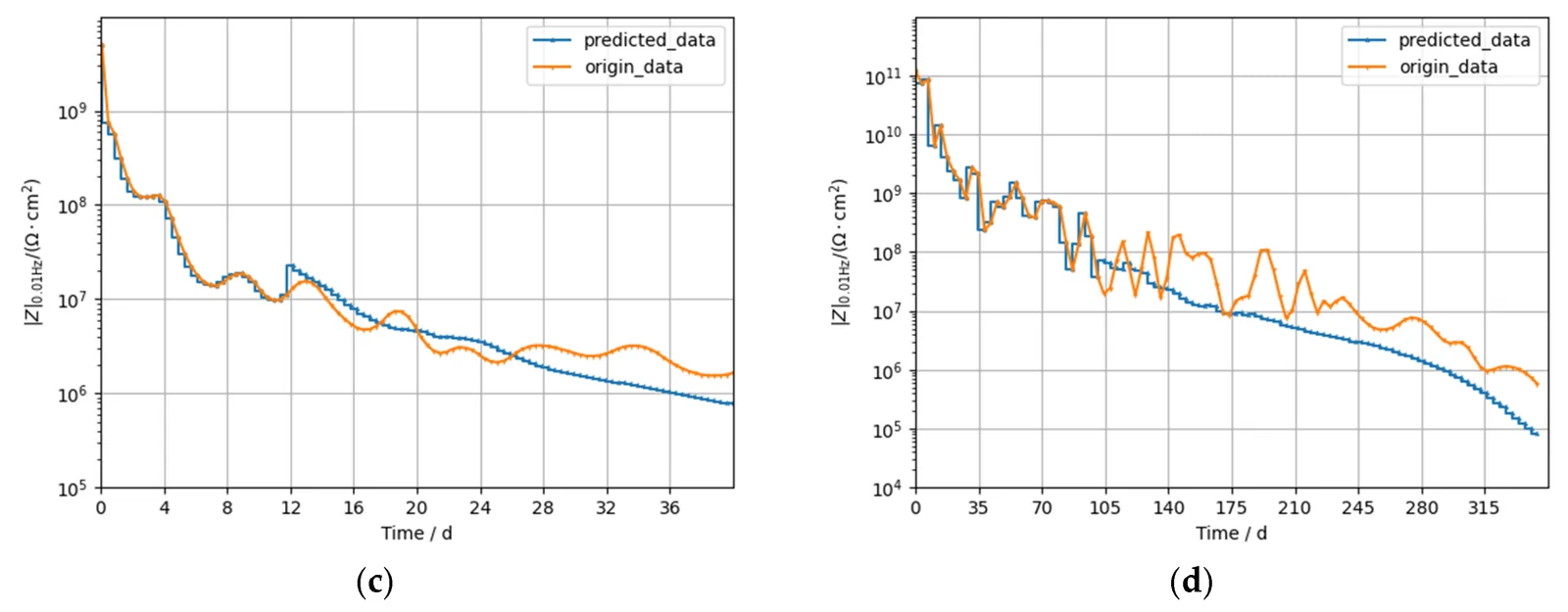
Comparison between model-predicted curves and actual coating performance evolution curves: (**a**) coating–environment system 1; (**b**) coating–environment system 4; (**c**) coating–environment system 6; (**d**) coating–environment system 7.

Four coating–environment combinations (1, 4, 6, and 7) from Table 1 are presented as representative examples. For each system, the parallel specimens were split into a model-building group and an independent checking group. The records from the model-building group were sorted chronologically by exposure time: the first 80% were used for model construction, and the subsequent 20% were reserved to verify model stability during training. The independent checking specimens belonged to the same coating–environment system, received no data augmentation, and remained completely unseen throughout model development. Figure 8 compares the predicted curves (blue lines) against the measured curves (yellow lines) obtained from this independent checking group. Trained on only early short-term monitoring data, the model successfully captured the nonlinear degradation trajectories, and the extrapolated curves showed close agreement with the subsequent measured data.

Quantitative comparisons between normalized predicted and measured curves are summarized in Table 3, which demonstrates the high predictive accuracy of the TET model across all experimental groups. Specifically, the cosine similarity exceeded 99.99% for all cases, verifying the model’s capability to faithfully reproduce the monotonic degradation trend of coating impedance. Meanwhile, all Euclidean distance values were lower than 0.01, indicating excellent performance in fitting the magnitude of impedance evolution. In this work, the critical low-frequency impedance modulus *Z*_crit_ (|*Z*|_0.01 Hz_) was adopted as the physical criterion for defining the coating end-of-service life to enable lifetime extrapolation. To guarantee physical plausibility, the group-specific *Z*_crit_ values listed in Table 3 were statistically determined from multi-dimensional experimental observables, including the onset of macroscopic blistering and rusting, substantial loss of adhesion, and the continuous negative shift in the open-circuit potential. These threshold values were further cross-validated against established failure criteria reported for epoxy-coated carbon steel systems in corrosive environments [13,21,26]. Notably, these impedance thresholds are system-dependent rather than universal constants. They are primarily valid for epoxy-based protective coatings on carbon steel substrates exposed to aqueous corrosive conditions and must be recalibrated for other coating–substrate combinations. Since the predicted remaining service life is highly sensitive to the predefined failure threshold, clarifying the physical origin and applicable scope of *Z*_crit_ is critical for reliable lifetime evaluation. Using these well-defined failure criteria, the relative errors of remaining-life predictions by the TET model ranged from 3.22% (Group 4) to 7.39% (Group 7). Such low prediction errors further confirm the high accuracy and reliability of the TET model for long-term coating lifetime prediction.

### 4.4. Comparative Experimental Design and Result Analysis

#### 4.4.1. Comparative Experimental Design

To validate the performance advantages of the TET model, comparative experiments were conducted against five mainstream time-series prediction models—traditional transformer, Informer, MLP (multilayer perceptron), LSTM and MLP-Mixer [28,29,30,31]. Four coating systems (No. 1, No. 2, No. 3 and No. 4) were selected from Table 1 for the experiments. Each dataset was represented by 30 consecutive monitoring points, with 300 parallel samples per group. All models were built and evaluated using the same preprocessed low-frequency impedance modulus sequences. Evaluation metrics employed were Euclidean distance (ED) and cosine similarity (CS): lower ED values indicated more accurate magnitude fitting, while CS values closer to one indicated higher similarity [32,33,34].

For each system, the model-building and independent checking specimens were strictly separated, and all models were examined on the independent checking specimens.

#### 4.4.2. Result Analysis and Discussion

(1) Prediction Accuracy and Error Performance:

The quantitative results presented in Table 4 demonstrate that the TET model exhibited excellent performance across most test scenarios. For coating system No. 1, No. 3 and No. 4, TET achieved both the smallest Euclidean distance and the highest cosine similarity, indicating superior comprehensive fitting capability. Notably, for coating system No. 1 and No. 3, its ED values were significantly lower than those of the other comparative models. Although the Euclidean distance of TET (1.4098) for coating system No. 2 was slightly higher than that of MLP-Mixer (1.2755), its cosine similarity remained at a high level of 0.9986.

(2) Comparison of Model Mechanisms and Limitations:

By incorporating time-embedding technology and a bidirectional LSTM module, the TET model achieved a favorable balance between efficiency and accuracy in processing long sequences. In contrast, other models exhibited certain limitations: the MLP structure was too simplistic to capture complex temporal features and was prone to overfitting, resulting in large errors (ED as high as 135–258); although LSTM could handle long-term dependencies, it was computationally intensive and susceptible to gradient issues; Informer, despite its advantages in long-sequence prediction, was sensitive to hyperparameter tuning; MLP-Mixer was better suited for image data and underperformed TET in long time-series tasks; the traditional transformer faced challenges with high computational complexity and potential overfitting. Overall, TET demonstrated a more balanced performance in terms of computational cost and prediction accuracy.

**Figure 9:**
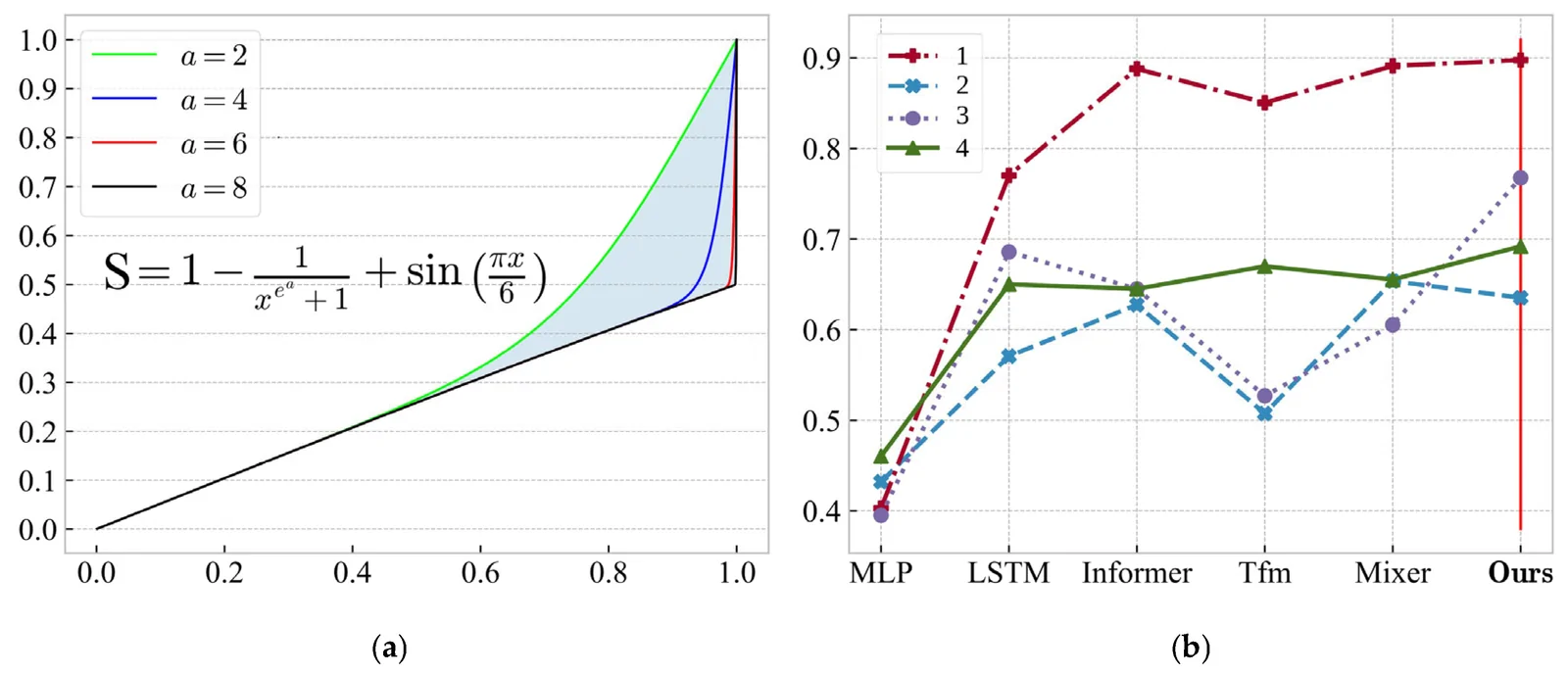
Analysis of S-function resolution enhancement: (**a**) schematic diagram of the S-function curve, (**b**) differential amplification effect of cosine similarity values from Table 4 after S-function transformation (*a* = 7.2), demonstrating the discriminability of model performance within the high-similarity region.

(3) Differential Analysis within High-Similarity Regions:

Given that the cosine similarity values of the advanced models in Table 4 were generally extremely high (>0.99), making subtle differences difficult to discern directly, a piecewise scaling function (Equation (10)) was introduced to enhance resolution in the critical region. By adjusting the scaling factor *α* (0.5 ≤ *α* ≤ 2.0), this function focused the evaluation on the high-similarity range of 0.99–1.0, thereby amplifying minor differences among the models. As shown in Figure 9, after processing with the S-function (with *α* = 7.2), the originally converging similarity values were effectively differentiated, clearly revealing the slight advantages of the TET model for coating system No. 1.

(10) $$S=\begin{array}{cc} 1-\frac{1}{x^{e^{a}}+1}+\sin\left(\frac{\mathrm{\pi x}}{6}\right) & a\in [1,10] \end{array}$$

**Figure 10:**
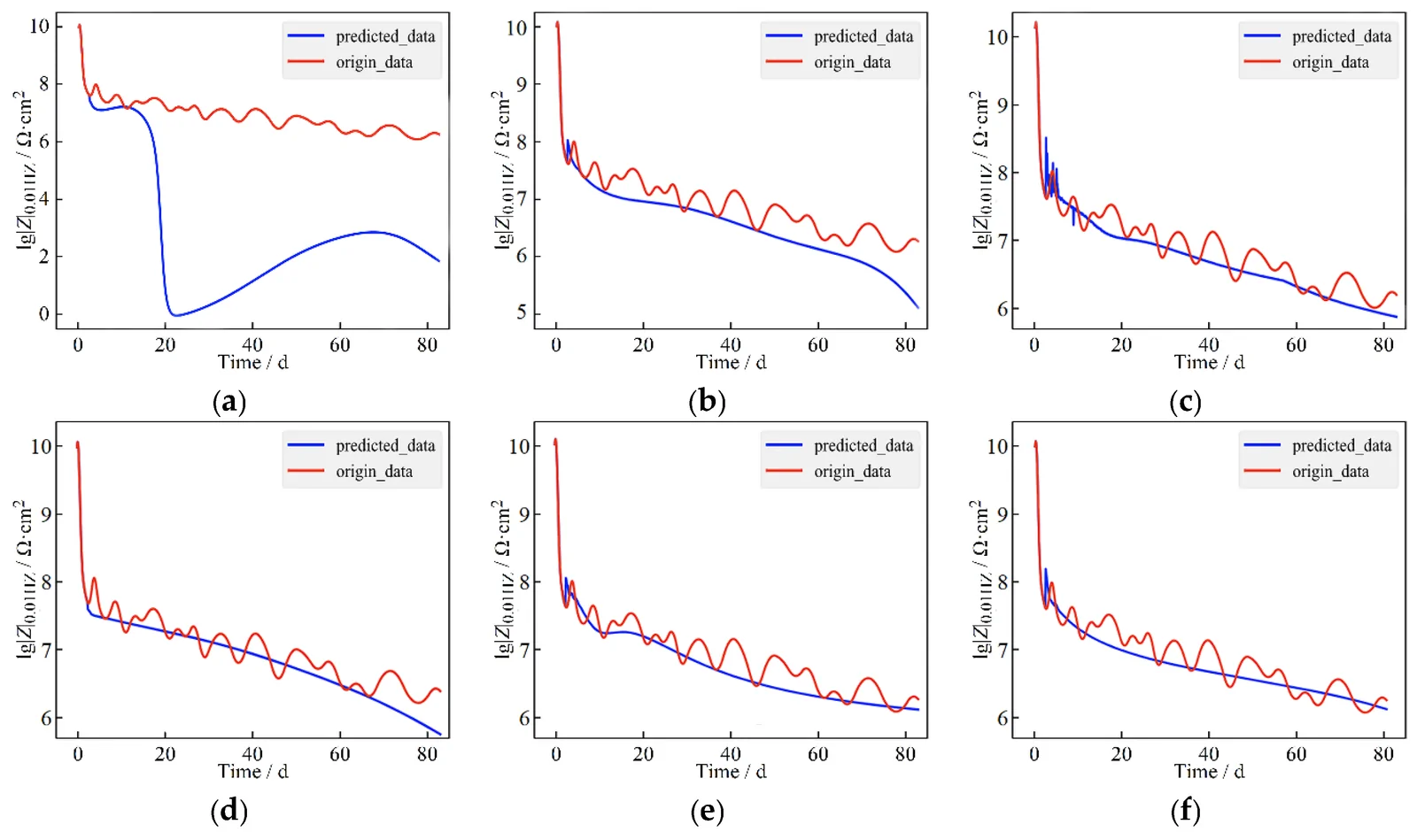
Comparison of model prediction results for coating system No. 1: (**a**) MLP, (**b**) LSTM, (**c**) Informer, (**d**) transformer, (**e**) MLP-Mixer, (**f**) TET.

Quantitative analysis (Table 4 and Figure 10f) shows that the TET model delivers the optimal overall performance in six out of eight dataset–metric comparisons (75%). TET yields the lowest Euclidean distance and the highest cosine similarity for three of the four evaluated systems. For coating system No. 1, TET produces an ED of 0.4133 and a CS of 0.99969, and its predicted curve closely tracks the monotonic decreasing trend of the measured data. Although MLP-Mixer exhibits a marginal advantage in one single metric for coating system No. 2, TET still retains a high cosine similarity of 0.99862. Across the four systems, TET achieves an average ED of 0.9243 and an average CS of 0.99912. These results demonstrate the strong application potential of the TET model for electrochemical lifetime prediction of coatings.

## 5. Conclusions

This study performed accelerated corrosion experiments on epoxy coatings under multiple corrosive conditions. Based on nine typical coating–environment combinations, the evolution of low-frequency impedance modulus (|*Z*|_0.01 Hz_) during coating aging was analyzed. The results demonstrate that this EIS indicator closely correlates with coating adhesion, open-circuit potential and macroscopic degradation morphology. The monotonic decline in |*Z*|_0.01 Hz_ across different coating systems and environments provides a reliable physical basis for characterizing coating degradation and achieving long-term lifetime extrapolation using short-term monitoring data.

To tackle the limited long-term extrapolation capability of conventional coating-degradation prediction approaches, this work proposes a time-embedding transformer (TET) model integrated with a time-embedding mechanism and a bidirectional LSTM module. The model can effectively capture long-range temporal dependencies within EIS sequences and suppress measurement noise, thereby addressing the limitations of conventional neural networks in learning dynamic degradation features and predicting long-term evolution trends. Independent blind validation verifies the predictive performance of the TET model. The cosine similarity between predicted and measured degradation curves exceeds 0.998 across all test cases. Compared with MLP, LSTM, Informer, transformer and MLP-Mixer, TET achieves the best overall performance for three out of the four evaluated systems (six of eight dataset–metric comparisons, 75%). The relative error in the predicted remaining service life of the coating stays below 8%. This study focuses on within-system evaluation. Cross-system transfer learning and explicit uncertainty quantification lie beyond the scope of the present dataset and are reserved as directions for future research.

The proposed TET framework can reliably extrapolate long-term coating-degradation behavior based on limited short-term EIS data, providing an accurate and feasible method for evaluating the remaining service life of organic anticorrosive coatings. This method supports coating durability assessment and maintenance decision-making for marine and aerospace corrosion protection. Future research will expand the dataset scale and working condition diversity. Enriching training data with various degradation patterns and environmental responses will further improve the model’s generalization and robustness, facilitating its extensive application in complex engineering scenarios.

## Figures and Tables

**Table 1:** Low-alloy steel/coating systems and corrosion test environments.

No.	Substrate (Pretreatment)	Coating Systems	Test Environments
1	Sa2.5	Solvent-free epoxy primer	3.5% NaCl solution (room temperature)
2			Neutral salt spray
3	St3		3.5% NaCl solution (room temperature)
4	Sa2.5	Waterborne epoxy primer	Neutral salt spray
5			3.5% NaCl solution (40 °C 12 h + 25 °C 12 h)
6	St3		3.5% NaCl solution (40 °C 12 h + 25 °C 12 h)
7	Sa2.5	Nano-modified epoxy primer	3.5% NaCl solution (room temperature)
8			Neutral salt spray
9			3.5% NaCl solution (40 °C 12 h + 25 °C 12 h)

**Table 2:** Adhesion, failure mode, and |*Z*|_0.01 Hz_ of nano-epoxy coating specimen at different experimental stages.

Time/d	|*Z*|_0.01 Hz_/Ω·cm^2^	Adhesion /MPa	Adhesion Attenuation Rate/%	Coating Failure Type and Area
0	1.1 × 10^11^	12.2	0	M/P: 0%; P:100%
77	1.3 × 10^8^	7.8	36.1	M/P: 5%; P: 95%
99	5.0 × 10^7^	6.5	40.6	M/P: 19%; P: 81%
260	1.0 × 10^7^	6.0	50.8	M/P: 46%; P: 54%
324	2.0 × 10^6^	4.7	61.5	M/P: 78%; P: 22%
370	1.0 × 10^6^	3.0	76.2	M/P: 94%; P: 6%

Note: P represents cohesive failure within the coating; P/M represents interfacial delamination at the coating/substrate interface.

**Table 3:** Quantitative metrics of TET model prediction performance on four representative coating–environment systems.

Performance Metrics	Parameter	Group 1	Group 4	Group 6	Group 7
Similarity	Cosine similarity	0.999996	0.999998	0.999992	0.999980
	Euclidean distance	0.003040	0.001902	0.003843	0.008345
Accuracy	|*Z*|_0.01 Hz_ threshold (Ω⋅cm^2^)	2 × 10^7^	6 × 10^5^	2 × 10^6^	1 × 10^6^
	Actual failure time (d)	66	47	38	345
	Predicted failure time (d)	68.3	48.5	39.2	319.5
	Lifetime prediction relative error (RE)	3.53%	3.22%	3.23%	7.39%

Note: Groups 1, 4, 6 and 7 match the identically numbered coating–environment systems in Table 1.

**Table 4:** Quantitative comparison results of model prediction performance.

Datasets	Metrics	MLP	LSTM	Informer	Tfm	Mixer	TET
Coating system No. 1	Euclidean	206.7330	1.8022	0.4869	0.6389	0.5392	0.4133
	Cosine	0.79170	0.99926	0.99966	0.99954	0.99967	0.99969
Coating system No. 2	Euclidean	187.7890	1.7460	1.4746	4.4223	1.2755	1.4098
	Cosine	0.85364	0.99809	0.99857	0.99650	0.99873	0.99862
Coating system No. 3	Euclidean	257.9240	1.3517	1.5178	2.9702	1.8751	0.9499
	Cosine	0.77540	0.99890	0.99868	0.99736	0.99841	0.99925
Coating system No. 4	Euclidean	135.2970	1.1268	1.5178	1.2244	1.0892	0.9243
	Cosine	0.91374	0.99871	0.99868	0.99882	0.99874	0.99893

Note: Tfm denotes transformer, Mixer denotes MLP-Mixer, and TET denotes time-embedding transformer. Coating system No. 1–4 correspond to sample sets 1–4 in Table 1.

## Data Availability

The original contributions presented in this study are included in the article. Further inquiries can be directed to the corresponding authors.
